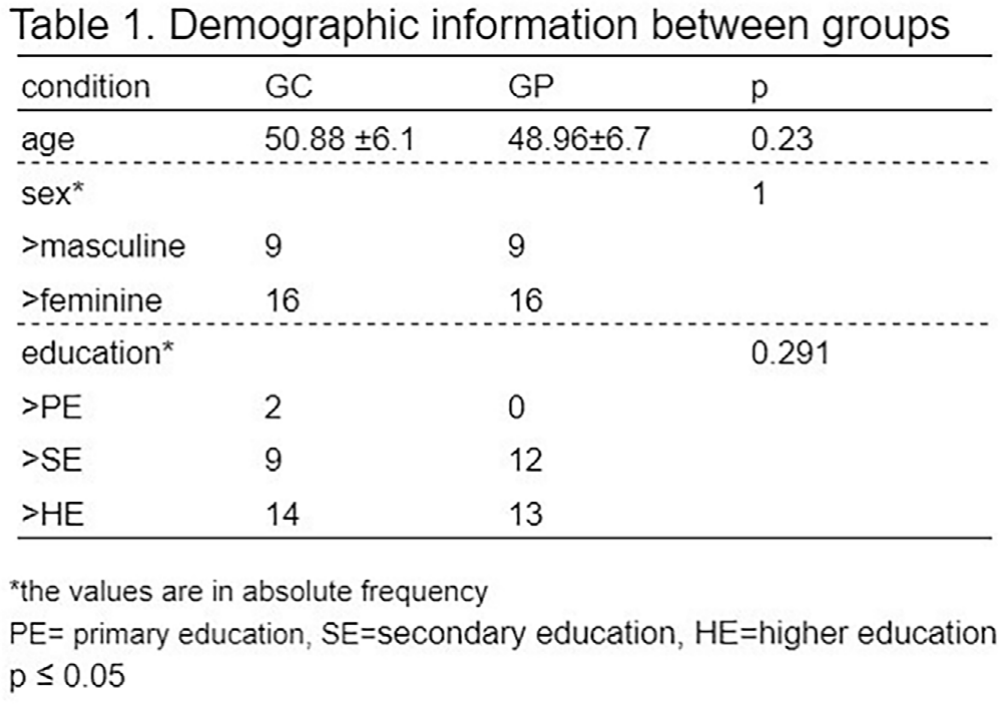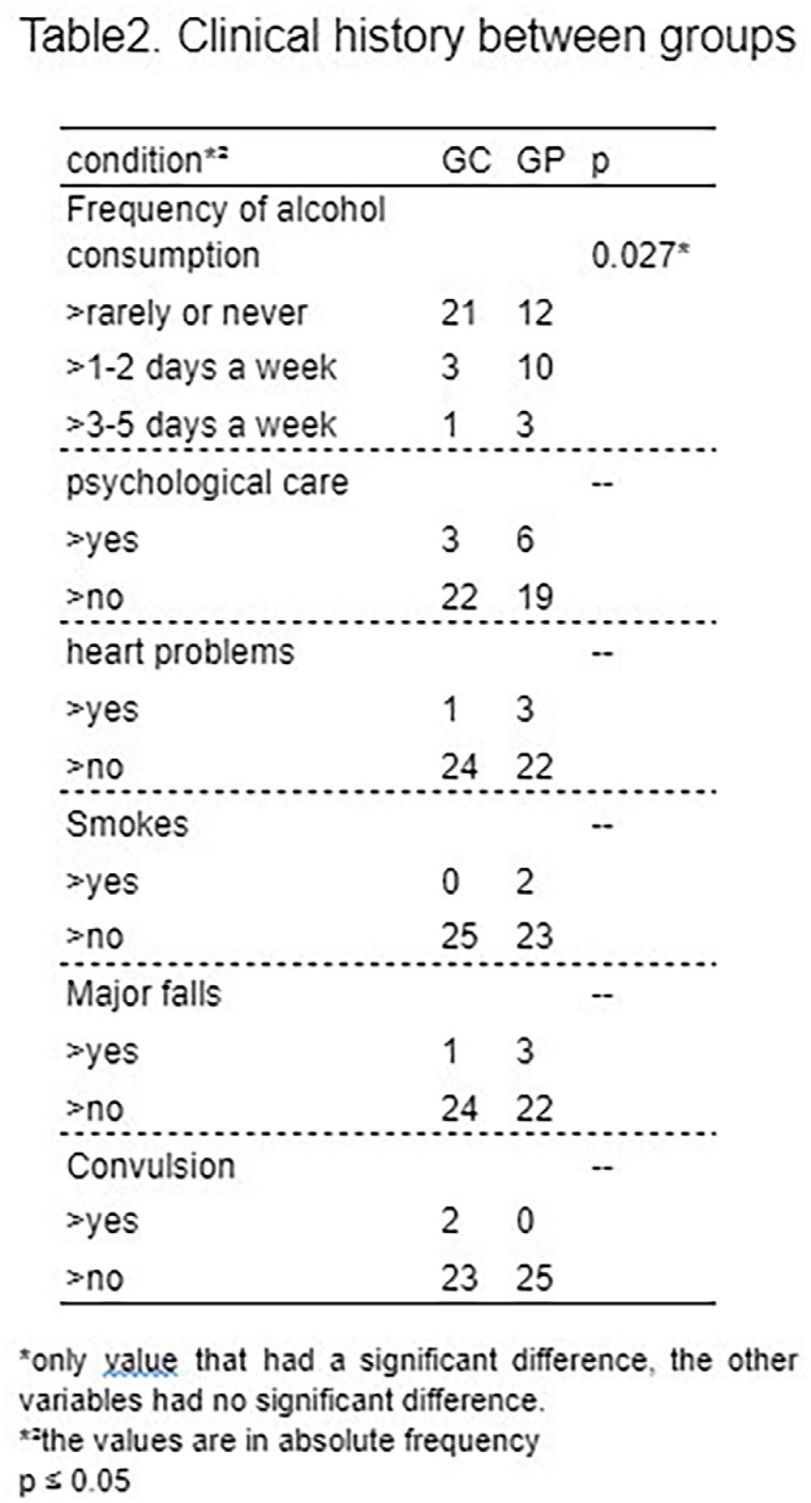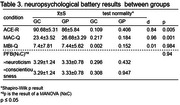# Predisposition to Alzheimer’s Disease in First‐Degree Relatives: Cognitive Decline, Personality Traits, and Prodromal Neuropsychiatric Symptoms

**DOI:** 10.1002/alz.087501

**Published:** 2025-01-03

**Authors:** Giovana Benassi Cezar, Marcos Josué Costa Dias, Adriana Oliveira Costa, Diego Alves Ferreira, Maria Paula Foss

**Affiliations:** ^1^ Hospital das Clínicas da Faculdade de Medicina da USP, Ribeirão Preto, São Paulo Brazil; ^2^ Faculdade de Filosofia, Ciências e Letras de Ribeirão Preto, USP, Ribeirão Preto, São Paulo Brazil; ^3^ School of Medicine of Ribeirão Preto, University of São Paulo, Ribeirão Preto, São Paulo Brazil; ^4^ School of Medicine of Ribeirão Preto, University of São Paulo, RIBEIRAO PRETO Brazil

## Abstract

**Background:**

Families with a history of Alzheimer’s Disease (AD) may have a genetic predisposition that raises the risk of developing the condition. However, not all members of these families can undergo genetic testing. Thus, this study aims to evaluate specific cognitive changes, neuropsychiatric symptoms (NPS), and personality traits that may act as early indicators of AD in family members compared to controls.

**Method:**

Fifty participants were divided into two groups for this study: cognitively healthy controls (CG; n = 25) and individuals with first‐degree relatives of AD (GP; n = 25). The education was represented as primary education (PE) from 1 to 9 years, secondary education (SE) from 10 to 12 years, and higher education (HE) from more than 13 years. Both groups underwent a neuropsychological battery (Adapted Background Questionnaire, Subjective Memory Assessment Clinics questionnaire (MAC‐Q), Addeenbroke’s cognitive examination revised (ACE‐R), Personality Factor Battery (PFB) and Mild Behavioral Impairment Checklist (MBI‐C)). Subsequently, the collected data was subjected to statistical analysis using a t‐test, MANOVA, and chi‐square, with a significance level of p≤0.05.

**Result:**

No significant differences between groups were found in age (p = 0.23), sex (p = 1.0), and education (p = 0.45) (Table 1). Except for alcohol consumption, there was no difference in medical history (Table 2). However, significant differences were encountered in ACE‐R (CG = 90.68±5.31; GP = 86±5.84; p = 0.005; d = 0.84) and MAC‐Q (GC = 23.4±3.52; GP = 26.68±3.29;p = 0.001;d = 0.96). In addition, no differences were demonstrated in MBI‐C (GC = 7.4±7.81; GP = 7.44±5.62;p = 0.984;d = 0.01) or the interaction between neuroticism (GC = 3.29±1.24;GP = 3.33±0.78) and conscientiousness (GC = 3.29±1.24;GP = 3.33±0.78;p = 0.94) in PFB (Table 3).

**Conclusion:**

Individuals who are first‐degree relatives of Alzheimer’s Disease patients tend to experience more cognitive complaints and show worse cognitive performance than controls. However, the two groups had no significant differences regarding NPS and personality traits. These findings were observed even after pairing the controls by sex, age, and education.